# Supplementary material for: Effects of core stability exercises on balance ability of children and adolescents with intellectual disabilities: A systematic review and meta-analysis
Source: PLoS One. 2024 Dec 19;19(12):e0314664. doi: 10.1371/journal.pone.0314664 (PMC11658597; doi:10.1371/journal.pone.0314664)
Supplement: S1 Table — (DOCX) [file pone.0314664.s001.docx]

Details of search query

| Database | Search queries |
| --- | --- |
| PubMed | (((core[Title/Abstract] OR “core stabili*”[Title/Abstract] OR “core training”[Title/Abstract] OR “core stabili* exercise”[Title/Abstract] OR “core stabili* training”[Title/Abstract] OR “core exercise”[Title/Abstract] OR “core strength*”[Title/Abstract] OR “Contraction of the abdominal muscles”[Title/Abstract]) AND (“mental* retardation”[Title/Abstract] OR “intelligence deficiency”[Title/Abstract] OR “intellectual impairment”[Title/Abstract] OR “mental barrier”[Title/Abstract] OR “mental* disorder”[Title/Abstract] OR “mental* development”[Title/Abstract] OR “intellectual development”[Title/Abstract] OR “intellectual disabilit*”[Title/Abstract] OR “cognitive impairment”[Title/Abstract] OR “Down Syndrome”[Title/Abstract] OR “Williams Syndrome”[Title/Abstract] OR “Prader Willi Syndrome”[Title/Abstract] OR “Rett syndrome”[Title/Abstract])) AND (child*[Title/Abstract] OR adolescent*[Title/Abstract] OR youth[Title/Abstract] OR teen*[Title/Abstract] OR pupil*[Title/Abstract] OR juvenile*[Title/Abstract] OR “school* aged”[Title/Abstract] OR student*[Title/Abstract] OR young[Title/Abstract])) AND (balance[Title/Abstract] OR “postural control”[Title/Abstract] OR “postural sway”[Title/Abstract] OR “postural stabili*”[Title/Abstract] OR “postural adaptation*”[Title/Abstract] OR “postural performance”[Title/Abstract] OR “postural perturbation”[Title/Abstract] OR “postural strateg*”[Title/Abstract] OR posture*[Title/Abstract] OR “postural balance”[Title/Abstract]) |
| WOS | (((TS=(core OR “core stabili*” OR “core training” OR “core stabili* exercise” OR “core stabili* training” OR “core exercise” OR “core strength* OR “Contraction of the abdominal muscles”)) AND TS=(“mental* retardation” OR “intellectual disabilit*” OR “intelligence deficiency” OR “intellectual impairment” OR “mental barrier” OR “mental* disorder” OR “mental* development” OR “intellectual development” OR “Down syndrome” OR “cognitive impairment” OR “Prader Willi Syndrome” OR “Rett syndrome” OR “Williams syndrome”)) AND TS=(child* OR adolescent* OR student* OR youth OR teen* OR pupil* OR juvenile* OR “school* aged” OR young)) AND TS=(balance OR “postural control*” OR “postural sway” OR “postural stabili*” OR “postural adaptation*” OR “postural performance” OR “postural perturbation” OR “postural strateg*” OR “posture*” OR “postural balance”) |
| Scopus | TITLE-ABS-KEY(core OR “core stabili*” OR “core training” OR “core stabili* exercise” OR “core stabili* training” OR “core exercise” OR “core strength*” OR “Contraction of the abdominal muscles”) AND TITLE-ABS-KEY(“mental* retardation” OR “intellectual disabilit*” OR “intelligence deficiency” OR “intellectual impairment” OR “mental barrier” OR “mental* disorder” OR “intellectual development” OR “mental* developmental” OR “Down syndrome” OR “cognitive impairment” OR “Prader Willi Syndrome” OR “Rett syndrome” OR “Williams syndrome”) AND TITLE-ABS-KEY(child* OR adolescent* OR student* OR youth* OR teen* OR pupil* OR juvenile* OR “school* aged” OR young) AND TITLE-ABS-KEY(balance OR “postural control*” OR “postural sway” OR “postural stabili*” OR “postural adaptation*” OR “postural performance” OR “postural perturbation” OR “postural strateg*” OR “posture*” OR “postural balance”) |
| Medline | SU core OR “core stabili*” OR “core training” OR “core stabili* exercise” OR “core stabili* training” OR “core exercise” OR “core strength*” OR “Contraction of the abdominal muscles” AND SU “mental* retardation” OR “intellectual disabilit*” OR “intelligence deficiency” OR “intellectual impairment” OR “mental barrier” OR “mental* disorder” OR “intellectual development” OR “mental* development” OR “Down syndrome” OR “cognitive impairment” OR “Prader Willi Syndrome” OR “Rett syndrome” OR “Williams syndrome” AND SU child* OR adolescent* OR student* OR youth* OR teen* OR pupil* OR juvenile* OR “school* aged” OR young AND SU balance OR “postural control*” OR “postural sway” OR “postural stabili*” OR “postural adaptation*” OR “postural performance” OR “postural perturbation” OR “postural strateg*” OR “posture*” OR “postural balance” |
| Embase | 'core':ab,ti OR 'core stabili*':ab,ti OR 'core training':ab,ti OR 'core stabili* exercise':ab,ti OR 'core stabili* training':ab,ti OR 'core exercise':ab,ti OR 'core strength*':ab,ti OR 'contraction of the abdominal muscles':ab,ti AND child*:ab,ti OR adolescent*:ab,ti OR youth:ab,ti OR teen*:ab,ti OR pupil*:ab,ti OR student*:ab,ti OR 'school* aged':ab,ti OR young:ab,ti AND 'postural balance':ab,ti OR 'postural control':ab,ti OR 'postural stabili*':ab,ti OR 'postural adaptation*':ab,ti OR 'postural performance':ab,ti OR 'postural strateg*':ab,ti OR 'postural sway':ab,ti OR posture*:ab,ti OR balance:ab,ti AND 'mental* retardation':ab,ti OR 'intellectual development':ab,ti OR 'intellectual disabilit*':ab,ti OR 'mental* disabilit*':ab,ti OR 'mental* development':ab,ti OR 'intelligence deficiency':ab,ti OR 'intelligence impairment':ab,ti OR 'mental* barrier':ab,ti OR 'mental* disorder':ab,ti OR 'cognitive impairment':ab,ti OR 'Down syndrome':ab,ti OR 'Prader Willi Syndrome':ab,ti OR 'Rett syndrome':ab,ti OR 'Williams syndrome':ab,ti |
| Cochrane Library | balance OR “postural control*” OR “postural sway” OR “postural stabili*” OR “postural adaptation*” OR “postural performance” OR “postural perturbation” OR “postural strateg*” OR posture* OR “postural balance” in Title Abstract Keyword AND core OR “core stabili*” OR “core training” OR “core stabili* exercise” OR “core stabili* training” OR “core exercise” OR “core strength*” OR “Contraction of the abdominal muscles” in Title Abstract Keyword AND “mental* retardation” OR “intellectual disabilit*” OR “intelligence deficiency” OR “intellectual impairment” OR “mental barrier” OR “mental disorder” OR “mental* development” OR “intellectual development” OR “Down syndrome” OR “cognitive impairment” OR “Prader Willi Syndrome” OR “Rett syndrome” OR “Williams syndrome” in Title Abstract Keyword AND child* OR adolescent* OR student* OR youth* OR teen* OR pupil* OR juvenile* OR “school* aged” OR young in Title Abstract Keyword |
