# Supplementary material for: Effects of core stability exercises on balance ability of children and adolescents with intellectual disabilities: A systematic review and meta-analysis
Source: PLoS One. 2024 Dec 19;19(12):e0314664. doi: 10.1371/journal.pone.0314664 (PMC11658597; doi:10.1371/journal.pone.0314664)
Supplement: S1 Appendix — (DOCX) [file pone.0314664.s003.docx]

## S3 Appendix. **List of excluded studies**

| **Reasons for exclusion** | **Amount** | **Study** |
| --- | --- | --- |
| **Interventions not based on core stability exercises** | **2** | **[1-2]** |
| **Non-English papers** | **6** | **[3-8]** |
| **Wrong outcome** | **3** | **[9-11]** |
| **Wrong publication type** | **16** | **[12-27]** |
| **Wrong study design** | **83** | **[28-110]** |
| **Wrong population** | **12** | **[111-122]** |

**References**

1. The effect of balance training on static and dynamic balance in children with intellectual disability. J Appl Environ Biol Sci. 2015;5(9):127-131.
2. Khaliltahmasebi R, Ghasemi G, Faramarzi S. The effects of rebound exercises on static and dynamic balance in educable children with intellectual disability. J Res Rehabil Sci. 2014;9(6).
3. Han K-H, Yun S. Effect of a 12-week core strengthening exercise program on the gait patterns and static balance ability of adolescents with intellectual disabilities. Sport Sci. 2023;41(2):21-8.
4. Lee C, Shin Doksu. Effects of core training on equilibrium sensory function, flexibility, lumbar muscle function and VAS in students with intellectual disability. Korean J Sport Sci. 2014;23(2):1379-88.
5. Im Jinseon, Jeong Yeonsu. Experience of caregiver supporting for independence living of the adults with intellectual disabilities. Korean J Parents and Guardians. 2018;5(2):87-105.
6. Daneshmandi H, Barati AH, Ahmadi R. The effect of core stabilization training program on the balance of mentally retarded educable students. Arch Rehabil. 2013;14(3):16-24.
7. Sayadinezhad T, Abdolvahab M, Akbarfahimi M, et al. The study of the effect of progressive resistance training on functional balance of 8-12 years old children with Down syndrome. J Modern Rehabil. 2013;7(1):29-33.
8. Lee Bomjin. Effects of core exercise on postures and physical fitness in students with intellectual disabilities. J Adapt Phys Act Exerc. 2016;24(4):1-12.
9. Sadredin S, Parivash S, Yousef Y. The effect and persistence of core stability training on balance in 10-16-year-old educable boys with intellectual disability. J Exceptional Children. 2017 Aug 10;17(1):53-64.
10. Balayi E, Sedaghati P. The effect of combined core stability and coordination exercises on the motor skills of intellectual disability with DCD. Phys Treatments. 2021;11(4):249-60.
11. Legerlotz K. The effects of resistance training on health of children and adolescents with disabilities. Am J Lifestyle Med. 2018 Feb 28;14(4):155982761875964.
12. Liu R, Li S, Guo R, Yu T, Dai Z, Zheng X, et al. Effect of Swiss ball training on abnormal plantar pressure distribution among adolescents with intellectual disabilities. Research Square. 2022 Mar 28.
13. Eggleston JD. Motor impairments in children with autism: Insight into the complexity of the disorder. 2018. (Note: This reference lacks a specific journal or publication title and may need further information for proper citation)
14. Ficicioglu C, Can K, Stepien KM. Alpha-Mannosidosis. In: Adam MP, et al., editors. GeneReviews®. Seattle (WA): University of Washington; 2001 Oct 11.
15. Goyal K, Goyal M, Samuel AJ. Core stabilization exercises impact on balance and foot pressure distribution in overweight children: A randomized controlled trial protocol. Rev Pesquisa Fisioterapia. 2020;10(4):724-36.
16. Haslam N. Introduction to personality and intelligence. 2007. (Note: This reference lacks a specific publication or book title and may need further information)
17. Jones P. Curricula for students with severe disabilities: narratives of standards-referenced good practice. New York: Routledge; 2017.
18. Nimmo DB. Mark Pattinson, the Pursuit of Learning, and the Modern University. Lancaster University, United Kingdom; 1979.
19. Schopp R, Wiener RL, Bornstein BH, Willborn SL, Springerlink. Mental Disorder and Criminal Law: Responsibility, Punishment and Competence. New York, Ny: Springer New York; 2009.
20. Urbano KV. Advances in genetics research. 2018.
21. Gupta S, Kovela RK. Effectiveness of dynamic neuromuscular stabilisation and neurodevelopmental therapy on gross motor function and trunk control in children with spastic diplegic cerebral palsy: A protocol for a RCT. J Clin Diagn Res. 2023;17(2):YK05-YK08.
22. Zhang M. The influence of core strength training on the physical quality of students with disabilities. In: 4th International Conference on Contemporary Education, Social Sciences and Humanities (ICCESSH 2019). Atlantis Press. 2019:1629-1634.
23. Chen ES. Studies on the implementation of backyard-style education under the safety management of schools. 2011.
24. Meagher K. Concepts of Art and Design Education: A Study of the Changing Curriculum with Special Reference to the Irish Education System [dissertation]. University of Liverpool; 2002.
25. Kim W-S. Basic research on the special dance education. Research of Dance Education. 2013
26. Dreismickenbecker E, Lanfranconi F, Stoessel S, et al. Get strong to fight childhood cancer: An exercise intervention for children and adolescents undergoing anti-cancer treatment (FORTEE). Pediatr Blood Cancer. 2022;69(Suppl 1). Published by Wiley, Hoboken, NJ, USA.
27. Knight A, Matheson M, Furth S, et al. Cognitive Function in Children with SLE Nephritis: A Cross-Sectional Comparison to Children with Other Glomerular Chronic Kidney Diseases. Arthritis Rheum. 2016;68(Suppl 10).
28. Aertssen WFM, Steenbergen B, Smits-Engelsman BCM. The validity and reliability of the Functional Strength Measurement (FSM) in children with intellectual disabilities. J Intellect Disabil Res. 2018;62(8):719-29.
29. Aggett P, Boyd E, Fletcher J. Developing a Tier I CAMHS foundation course: Report on a 4-year initiative. Clin Child Psychol Psychiatry. 2006;11(3):319-33.
30. Angelova S, Spassov B, Nikolova V, Christov I, Tzvetkov N, Simeonova M. Is the amplification of c-MYC, MLL and RUNX1 genes in AML and MDS patients with trisomy 8, 11 and 21 a factor for a clonal evolution in their karyotype? Tsitologiia i genetika. 2015;49(3):25-32.
31. Anugrah T, Simatupang N, Sutapa P, Ambardini RL, Nugroho S. The relationship of dynamic body balance with locomotor ability and endurance of core muscles in children with Down syndrome. J Kinesiol Exerc Sci. 2023;33(102):1-8.
32. Bernardo P, Cobb S, Coppola A, Tomasevic L, Di Lazzaro V, Bravaccio C, et al. Neurophysiological signatures of motor impairment in patients with Rett syndrome. Ann Neurol. 2020;87(5):763-73.
33. Burke LW, Wiley JE, Glenn CC, Driscoll DJ, Loud KM, Smith AJ, et al. Familial cryptic translocation resulting in Angelman syndrome: implications for imprinting or location of the Angelman gene? Am J Hum Genet. 1996;58(4):777-84.
34. Casey J, Jenkinson A, Magee A, Ennis S, Monavari A, Green A, et al. Beaulieu-Boycott-Innes syndrome: An intellectual disability syndrome with characteristic facies. Clin Dysmorphol. 2016;25(4):146-51.
35. Choi JY, Ha SW, Jeong D-E, Lee J, Kim D, Min J-Y, et al. Association between the loss of gait harmony and cognitive impairment: Cross-sectional study. JMIR Public Health Surveill. 2023;9.
36. Chopra M, McEntagart M, Clayton-Smith J, Platzer K, Shukla A, Girisha KM, et al. Heterozygous ANKRD17 loss-of-function variants cause a syndrome with intellectual disability, speech delay, and dysmorphism. Am J Hum Genet. 2021;108(6):1
37. De Lumley L, Vallat JM, Catanzano G. Clinical and ultrastructural study of a case of congenital myopathy with multiple foci. Semaine des Hopitaux. 1976 Sep 26;52(37):733-6.
38. Dellve L, Cernerud L, Hallberg LR. Harmonizing dilemmas: Siblings of children with DAMP and Asperger syndrome's experiences of coping with their life situations. Scandinavian Journal of Caring Sciences. 2000;14(3):172-178.
39. Deutsch SI, Urbano MR, Neumann SA, Burket JA, Katz E. Cholinergic Abnormalities in Autism: Is There a Rationale for Selective Nicotinic Agonist Interventions? Clinical Neuropharmacology. 2010 Sep;33(3):114-120.
40. Dodd DCH, Zabriskie RB, Widmer MA, Eggett D. Contributions of Family Leisure to Family Functioning Among Families that Include Children with Developmental Disabilities. Journal of Leisure Research. 2009;41(2):261-286.
41. Dondorp W, de Wert G, Bombard Y, Bianchi DW, Bergmann C, Borry P, et al. Non-invasive prenatal testing for aneuploidy and beyond: challenges of responsible innovation in prenatal screening. European Journal of Human Genetics. 2015 Nov;23(11):1438-1450.
42. Donoso FA, Arriagada SD, Diaz RF, Cruces RP. Invasive mechanical ventilation: Update for the pediatrician. Archivos Argentinos de Pediatria. 2013 Sep;111(5):428-436.
43. Duchesne A, Vaiman A, Frah M, Floriot S, Legoueix-Rodriguez S, Desmazieres A, et al. Progressive ataxia of Charolais cattle highlights a role of KIF1C in sustainable myelination. PLoS Genetics. 2018 Aug 2;14(8):e1007574.
44. Eldal K, Natvik E, Veseth M, Davidson L, Skjølberg Å, Gytri D, et al. Being recognised as a whole person: A qualitative study of inpatient experience in mental health. Issues in Mental Health Nursing. 2019 Mar;40(2):88-96.
45. Fulceri F, Grossi E, Contaldo A, Narzisi A, Apicella F, Parrini I, et al. Motor skills as moderators of core symptoms in autism spectrum disorders: Preliminary data from an exploratory analysis with artificial neural networks. Frontiers in Psychology. 2019 Jan;9:2766.
46. Hilgarter K, Schmid-Zalaudek K, Csanady-Leitner R, Moertl M, Roessler A, Lackner HK. Phasic heart rate variability and the association with cognitive performance: A cross-sectional study in a healthy population setting. PLoS One. 2021 Mar 16;16(3):e0248633.
47. Hojlo MA, Milliken AL, Baumer NT, Davidson EJ. DSFit: a feasibility pilot study of a group exercise programme for adolescents with Down syndrome. Journal of Intellectual Disability Research. 2022 Dec;66(12):952-966.
48. Ikeda M. Regional network for patients with dementia--carrying out Kumamoto model for dementia. Seishin Shinkeigaku Zasshi (Psychiatria et Neurologia Japonica). 2014 Oct;116(5):395-400.
49. Jain PD, Nayak A, Karnad SD. Relationship between trunk muscle strength, reaching ability, and balance in children with Down syndrome: A cross-sectional study. Brain and Development. 2022 Mar;44(2):95-104.
50. Jans T, Ball J, Preiss M, Haberhausen M, Warnke A, Renner TJ. Pervasive refusal syndrome: Three German cases provide further illustration. Zeitschrift für Kinder- und Jugendpsychiatrie und Psychotherapie. 2011 Sep;39(5):351-359.
51. Kaltiala-Heino R, Kahila K. Forensic psychiatric inpatient treatment: Creating a therapeutic milieu. Child and Adolescent Psychiatric Clinics of North America. 2006 Jun;15(2):459-475.
52. Kuntz EM, Carter EW. Review of Interventions Supporting Secondary Students with Intellectual Disability in General Education Classes. Research and Practice for Persons with Severe Disabilities. 2019 Spring;44(2):103-121.
53. Kuslich CD, Kobori JA, Mohapatra G, Gregorio-King C, Donlon TA. Prader-Willi syndrome is caused by disruption of the SNRPN gene. American Journal of Human Genetics. 1999 Jul;64(1):70-76.
54. Lord C, Jones RM. Annual Research Review: Re-thinking the classification of autism spectrum disorders. Journal of Child Psychology and Psychiatry. 2012 Apr 4;53(5):490–509.
55. Ma Y, Wang L, Li M, Wang T. Meta-analysis of the effects of exercise programs in improving the balance ability of children with intellectual disabilities. Journal of Intellectual & Developmental Disability. 2020 Mar;45(2):144-154.
56. Ma Y, Zhang K, Li S, Wang L, Wang T. Biomechanical analysis of gait patterns in children with intellectual disabilities. Journal of Intellectual Disability Research. 2021 Oct;65(10):912-921.
57. Maresca G, Nocito V, De Salvo S, Formica C, Leonardi S, Bramanti P, et al. Assessment and rehabilitation of cognitive deficit in a Niemann-Pick type C disease patient. Neurocase. 2021 Mar;27(2):113-116.
58. Moke L, Severijns P, Schelfaut S, Van de Loock K, Hermans L, Molenaers G, et al. Performance on Balance Evaluation Systems Test (BESTest) Impacts Health-Related Quality of Life in Adult Spinal Deformity Patients. Spine. 2018 Apr 15;43(9):637-646.
59. Moy SS, Nadler JJ, Young NB, Nonneman RJ, Grossman AW, Murphy DL, et al. Social approach in genetically engineered mouse lines relevant to autism. Genes, Brain, and Behavior. 2009 Apr;8(2):129-142.
60. Munde V, Vlaskamp C. Initiation of activities and alertness in individuals with profound intellectual and multiple disabilities. Journal of Intellectual Disability Research. 2015 Sep;59(3):284-292.
61. Munjas BA. Chronicity in mental illness: Does "nursing core" maintain it? Issues in Mental Health Nursing. 1980 Sep;2(3):1-13.
62. Nomura Y, Segawa M. Characteristics of motor disturbances of the Rett syndrome. Brain & Development. 1990;12(1):27-30.
63. Osborne KJ, Zhang W, Farrens J, Geiger M, Kraus B, Glazer J, et al. Neural Mechanisms of Motor Dysfunction in Individuals at Clinical High-Risk for Psychosis: Evidence for Impairments in Motor Activation. Journal of Psychopathology and Clinical Science. 2022;131(4):375-391.
64. Pang W, Yi X, Li L, Liu L, Xiang W, Xiao L. Untangle the Multi-Facet Functions of Auts2 as an Entry Point to Understand Neurodevelopmental Disorders. Frontiers in Psychiatry. 2021;12.
65. Purpura G, Fulceri F, Puglisi V, Masoni P, Contaldo A. Motor coordination impairment in children with autism spectrum disorder: A pilot study using Movement Assessment Battery for Children-2 Checklist. Minerva Pediatrica. 2020;72(1):22-29.
66. Radonovich KJ, Fournier KA, Hass CJ. Relationship between postural control and restricted, repetitive behaviors in autism spectrum disorders. Frontiers in Integrative Neuroscience. 2013;7:28.
67. Raggi A, Covelli V, Schiavolin S, Scaratti C, Leonardi M, Willems M. Work-related problems in multiple sclerosis: a literature review on its associates and determinants. Disability and Rehabilitation. 2016;38(10):936-944.
68. Razak F, Smith GD, Subramanian SV. The idea of uniform change: is it time to revisit a central tenet of Rose's "Strategy of Preventive Medicine"? American Journal of Clinical Nutrition. 2016;104(6):1497-1507.
69. Rodriguez-Grande E-I, Vargas-Pinilla O-C, Torres-Narvaez M-R, Rodriguez-Malagon N. Neuromuscular exercise in children with Down Syndrome: a systematic review. Scientific Reports. 2022;12(1).
70. Sahoo T, del Gaudio D, German JR, Shinawi M, Peters SU, Person RE, et al. Prader-Willi phenotype caused by paternal deficiency for the HBII-85C/D box small nucleolar RNA cluster. Nature Genetics. 2008;40(6):719-721.
71. Schulze A, Hansen C, Skakkebaek NE, Brondum-Nielsen K, Ledbeter DH, Tommerup N. Exclusion of SNRPN as a major determinant of Prader-Willi syndrome by a translocation breakpoint. Nature Genetics. 1996;12(4):452-454.
72. Sharma A, Gokulchandran N, Kulkarni P, Kiran Mullangi S, Bhagawanani K, Ganar V, et al. Multiple cellular therapies along with neurorehabilitation in spastic diplegic cerebral palsy: A case report. Innovations in Clinical Neuroscience. 2020;17(10-12):31-34.
73. Sorellad S, Lapomarda G, Messina I, Frederickson JJ, Siugzdaite R, Job R, et al. Testing the expanded continuum hypothesis of schizophrenia and bipolar disorder. Neural and psychological evidence for shared and distinct mechanisms. Neuroimage Clinical. 2019;23.
74. Sukriadi S, Setiakarnawijaya Y, Tangkudung J, Dlis F, Dwi DRAS, Arif M, et al. Analysis of Physical Fitness Profile of Athletes with Intellectual Disability in Special Olympics Indonesia (SOIna) after the Covid-19 Pandemic. International Journal of Human Movement and Sports Sciences. 2023;11(5):1044-1059.
75. Sun Y, Nicholls RD, Butler MG, Saitoh S, Hainline BE, Palmer CG. Breakage in the SNRPN locus in a balanced 46,XY,t(15;19) Prader-Willi syndrome patient. Human Molecular Genetics. 1996;5(4):517-524.
76. Trzepacz PT, Meagher DJ, Franco JG. Comparison of diagnostic classification systems for delirium with new research criteria that incorporate the three core domains. Journal of Psychosomatic Research. 2016;84:60-68.
77. Uhlhaas PJ, Gajwani R, Gross J, Gumley AI, Lawrie SM, Schwannauer M. The Youth Mental Health Risk and Resilience Study. BMC Psychiatry. 2017;17(1).
78. Vandewalle J, Beeckman D, Van Hecke A, Debyser B, Deproost E, Verhaeghe S. ‘Promoting and preserving safety and a life-oriented perspective’: A qualitative study of nurses’ interactions with patients experiencing suicidal ideation. International Journal of Mental Health Nursing. 2019;28(5):1119-1131.
79. Ventola P, Pomales-Ramos A, Delucia EA. Longitudinal cognitive and behavioral presentation of adult female with kabuki syndrome. American Journal of Case Reports. 2019;20:430-436.
80. Vukadin L, Kim J-H, Hackwelder TB, Ungerleider N, Flemington E, Ahn E-YE. Hematological Disorders in Human Patients with Son Mutations. Blood. 2019;134.
81. Wang J-C, Vaccarello-Cruz M, Ross L, Owen R, Pratt VM, Lightman K, et al. Mosaic isochromosome 15q and maternal uniparental isodisomy for chromosome 15 in a patient with morbid obesity and variant PWS-like phenotype. American Journal of Medical Genetics Part A. 2013;161(7):1695-1701.
82. Wayne DOM, Krishnagiri S. Parents' leisure: the impact of raising a child with Down syndrome. Occupational Therapy International. 2005;12(3):180-194.
83. Weiss B. Endocrine Disruptors as a Factor in Mental Retardation. International Review of Research in Mental Retardation. 2005;30:195-223.
84. Zollino M, Murdolo M, Marangi G, Pecile V, Galasso C, Mazzanti L, et al. On the Nosology and Pathogenesis of Wolf-Hirschhorn Syndrome: Genotype-Phenotype Correlation Analysis of 80 Patients and Literature Review. American Journal of Medical Genetics Part C: Seminars in Medical Genetics. 2008;148(4):257-269.
85. Ely BR, Sollanek KJ, Cheuvront SN, Lieberman HR, Kenefick RW. Hypohydration and acute thermal stress affect mood state but not cognition or dynamic postural balance. European Journal of Applied Physiology. 2012 Oct 12;113(4):1027-1034.
86. Carvalho RL, Almeida GL. The effect of vibration on postural response of Down syndrome individuals on the seesaw. Research in Developmental Disabilities. 2009 Nov;30(6):1124-1131.
87. Eid MA. Effect of Whole-Body Vibration Training on Standing Balance and Muscle Strength in Children with Down Syndrome. American Journal of Physical Medicine & Rehabilitation. 2015 Aug;94(8):633-643.
88. Song HS, Kim JY, Park SD. Effect of the class and individual applications of task-oriented circuit training on gait ability in patients with chronic stroke. Journal of Physical Therapy Science. 2015;27(1):187-189.
89. Green J, Leadbitter K, Ellis C, et al. Combined social communication therapy at home and in education for young autistic children in England (PACT-G): a parallel, single-blind, randomised controlled trial. The Lancet Psychiatry. 2022;9(4):307-320
90. Ma S. RESEARCH ON THE INFLUENCE OF THE DEVELOPMENT OF COLLEGE STUDENT ASSOCIATIONS ON THE ALLEVIATION OF COLLEGE STUDENTS’MENTAL STRESS IN THE NEW PERIOD. Psychiatria Danubina. 2021, 33(suppl 8): 93-95.
91. Alhasani R. The effect of a novel dual-task exercise program for balance, mobility, gaze, and cognition skills in community dwelling older adults: A pilot study. 2015.
92. West AE, Weinstein SM, Pavuluri MN. Family-based cognitive-behavioural therapy for paediatric bipolar disorder. Int Clin Psychopharmacol. 2011;26:e34-e35.
93. Wolkenstein L, Zeiller M, Kanske P, Plewnia C. Induction of a depression-like negativity bias by cathodal transcranial direct current stimulation. Cortex. 2014;59:103-112.
94. Van de Winckel A, Carpentier S, Deng W, et al. Identifying Body Awareness-Related Brain Network Changes After Cognitive Multisensory Rehabilitation for Neuropathic Pain Relief in Adults With Spinal Cord Injury: Protocol of a Phase I Randomized Controlled Trial. Top Spinal Cord Inj Rehabil. 2022;28(4):33-43.
95. Lam AP, Matthies S, Graf E, et al. Long-term Effects of Multimodal Treatment on Adult Attention-Deficit/Hyperactivity Disorder Symptoms. JAMA Netw Open. 2019;2(5):e194980.
96. Bezner JR, Lloyd L, Crixell S, Franklin K. Health behaviour change coaching in physical therapy: improving physical fitness and related psychological constructs of employees in a university setting. Eur J Physiother. 2017;19(Suppl 1):1-2.
97. Haugstad GK, Kirste U, Leganger S, et al. Somatocognitive therapy in the management of chronic gynaecological pain. A review of the historical background and results of a current approach. Scand J Pain. 2011;2(3):124-129.
98. Wang Y, Tong L, Xiong S, et al. STUDY ON THE STRATEGY OF RELIEVING CHILDREN’S ANXIETY IN THE TREATMENT OF CHILDREN WITH KAWASAKI DISEASE. Psychiatria Danubina. 2022;34(Suppl 2):20.
99. Yang G. APPLICATION OF THINKING LOGIC BARRIER ANALYSIS IN OPTIMIZATION OF TEACHING MATERIALS AND METHODS OF MATHEMATICS EDUCATION. Psychiatria Danubina. 2021;33(7):433-434.
100. Ma Y, Wang L, Li M, Wang T. Meta-analysis of the effects of exercise programs in improving the balance ability of children with intellectual disabilities. J Intellect Dev Disabil. 2019;Aug 8: 11.
101. Bahiraei S, Firouzjah EN, Abbaszadeh A. Exercise training to improve balance ability for individuals with Down syndrome: a systematic review and meta-analysis. Adv Rehabil. 2023;37(1):12-22.
102. Blomqvist S, Lönnberg L, Sundelin G, et al. Physical Exercise Frequency Seem not to Influence Postural Balance but Trunk Muscle Endurance in Young Persons with Intellectual Disability. J Phys Educ Sports Manag. 2017;4(2).
103. Haghighi AH, Mohammadtaghipoor F, Hamedinia M, et al. Effect of a combined exercise program (aerobic and rebound therapy) with two different ratios on some physical and motor fitness indices in intellectually disabled girls. Baltic J Health Phys Act. 2019;11(1):3.
104. Haynes H. Physiotherapy for adolescents with developmental disabilities who require secure care. In: Forensic Issues in Adolescents with Developmental Disabilities. Edited by Jessica Kingsley Publishers, London. 2011;127-141.
105. Zolghadr H, Sedaghati P, Daneshmandi H. The Effect of Selected Balance/Corrective Exercises on the Balance Performance of Mentally-Retarded Students With Developmental Coordination Disorder. Phys Treat Spec Phys Ther J 2019;23-30.
106. Adeeb N, Farooqui SI, Khan A, Rizvi J, Kazmi SAM. Current Approaches to Improve Balance in Down Syndrome Population-A Systematic Review. J Intellect Disabil-Diagn Treat. 2021;9(3):195-205.
107. Benedetti MG, Berti L, Presti C, et al. Effects of an adapted physical activity program in a group of elderly subjects with flexed posture: clinical and instrumental assessment. J NeuroEng Rehabil. 2008;5(1).
108. Boer PH. The effect of 8 weeks of freestyle swim training on the functional fitness of adults with Down syndrome. J Intellect Disabil Res. 2020;64(10).
109. Kang OD. Effects of therapeutic horse-riding program on the walking ability of students with intellectual disabilities. J Anim Sci Technol. 2021;63(2):440.
110. Stier-Jarmer M, Throner V, Kirschneck M, et al. The psychological and physical effects of forests on human health: A systematic review of systematic reviews and meta-analyses. Int J Environ Res Public Health. 2021;18(4):1770.
111. Barone R, Sturiale L, Fiumara A, Palmigiano A, Bua RO, Rizzo R, et al. CSF N-glycan profile reveals sialylation deficiency in a patient with GM2 gangliosidosis presenting as childhood disintegrative disorder. Autism Res. 2016;9(4):423-8.
112. Del Bianco T, Ozturk Y, Basadonne I, Mazzoni N, Venuti P. The Thorn in the Dyad: A Vision on Parent-Child Relationship in Autism Spectrum Disorder. Eur J Psychol. 2018;14(3):695-709.
113. Munesue T, Nakamura H, Kikuchi M, Miura Y, Takeuchi N, Anme T, et al. Oxytocin for Male Subjects with Autism Spectrum Disorder and Comorbid Intellectual Disabilities: A Randomized Pilot Study. Front Psychiatry. 2016;7:165.
114. Usama M, Abdelaziem F, Rashed WM, Maher E, El Beltagy M, Zekri W. Impact of physical activity on postural stability and coordination in children with posterior fossa tumor: Randomized control phase III trial. J Cancer Res Clin Oncol. 2023;149(9):5637-44.
115. Zarei H, Ali AN. Effects of proprioception and core stability training followed by detraining on balance performance in deaf male students: A three-arm randomized controlled trial. Somatosens Mot Res. 2023;40(2):47-55.
116. Rahmayanti A, Rahman F, Herlinawati I. The Effect of Combination of Core Stability Training and Lower Extremity Strength Training on Static Standing Balance Ability in a Child with Down Syndrome. J Adv Multidiscip Res. 2022;3(2):71-78.
117. Javadipour Z, et al. The Effects of Combining Core Stability with Stretching Exercises on Pain Intensity and Motor Function in People with Chronic Nonspecific Low Back Pain with and without Hyperlordosis. Ann App Sport Sci. 2021;9(2):59-67.
118. Kahle NL, Gribble PA. Core Stability Training in Dynamic Balance Testing among Young, Healthy Adults. Athl Train Sports Health Care. 2009;1(2):65-73.
119. Sakzewski L, Bleyenheuft Y, Novak I, et al. HABIT-ILE Australia: Randomised trial of Hand-Arm Bimanual Intensive Training Including Lower Extremities for children with bilateral cerebral palsy. Dev Med Child Neurol. 2024;66(2):49.
120. Chung EJ, Kim JH, Lee BH. The Effects of Core Stabilization Exercise on Dynamic Balance and Gait Function in Stroke Patients. J Phys Ther Sci. 2013;25(7):803-6.
121. Gheitasi M, Bayattork M, Miri H, Afshar H. Comparing the Effect of Suspended and Non-Suspended Core Stability Exercises on Static and Dynamic Balance and Muscular Endurance in Young Males With Down Syndrome. Phys Treat Spec Phys Ther J. 2019;15:153-60.
122. Omidi M, Shamsi Majalan A, Karimizadeh Ardakani M, Hani Mansoori M. The Effect of a 6-week Core Stability Exercises on the Risk of Falling and Quality of Life in Blind People. Phys Treat Spec Phys Ther J. 2019;15:227-34.
