# Supplementary material for: Effects of core stability exercises on balance ability of children and adolescents with intellectual disabilities: A systematic review and meta-analysis
Source: PLoS One. 2024 Dec 19;19(12):e0314664. doi: 10.1371/journal.pone.0314664 (PMC11658597; doi:10.1371/journal.pone.0314664)
Supplement: Attachment — Submitted filename: PONE-D-24-21189.pdf [file pone.0314664.s005.pdf]

PLOS ONE

Effects of core stability exercises on balance ability of children and adolescents with intellectual disabilities: A systematic review and meta-analysis

--Manuscript Draft--

Manuscript Number:	PONE-D-24-21189
Article Type:	Research Article
Full Title:	Effects of core stability exercises on balance ability of children and adolescents with intellectual disabilities: A systematic review and meta-analysis
Short Title:	Effects of core stability exercises on balance ability of youth with intellectual disabilities
Corresponding Author:	wenhong xu<br>Zhejiang Normal University<br>Jinh, CHINA
Keywords:	core stability exercises; intellectual disabilities; Meta-analysis; balance ability; children; adolescents
Abstract:	Background<br>In recent years, core stability exercises have been of interest to researchers in improving the balance functions of children and adolescents with disabilities. Studies show that children and adolescents with intellectual disabilities (ID) have more adverse balance and less muscle strength than typically developing peers.<br>Objective<br>This systematic review and meta-analysis aim to summarise and quantify the effectiveness of core stability exercises on improving the balance of this target population.<br>Methods<br>English-language articles were searched up to June 2023 from 6 electronic databases. Two independent authors assessed the risk of bias using the Cochrane Collaboration's risk of bias tool. Meta-analysis was performed using the RevMan 5.4.<br>Results<br>A total of six studies were included, four of which contributed information to the meta-analysis. Our narrative synthesis revealed that core stability exercises improve the dynamic balance of children and adolescents with ID. The pooled analysis shows that core stability exercises were effective for static (Hedges' g: 1.32; 95%CI = -0.41 to 3.06; p = .05) and static-dynamic (Hedges' g: 1.35; 95%CI = -0.02 to 2.73; p = .13) balance in children and adolescents with ID, but the effect was insignificant compared with their peers in the control group.<br>Conclusions<br>Core stability exercises are effective in improving dynamic balance in children and adolescents with ID. However, no significant effects were found on the improvement in static and static-dynamic balance in this population. More randomized controlled trials are needed to verify the findings of the current study.
Order of Authors:	Junjie Zhou
	Yecheng Zhong
	Wenhong Xu
Additional Information:	
**Question**	**Response**
**Financial Disclosure**<br><br>Enter a financial disclosure statement that describes the sources of funding for the work included in this submission. Review the submission guidelines for detailed requirements. View published research	The author(s) received no specific funding for this work.

Powered by Editorial Manager® and ProduXion Manager® from Aries Systems Corporation

articles from *PLOS ONE* for specific examples.<br><br>This statement is required for submission and **will appear in the published article** if the submission is accepted. Please make sure it is accurate.<br><br>**Funded studies**<br>Enter a statement with the following details:<br>• Initials of the authors who received each award<br>• Grant numbers awarded to each author<br>• The full name of each funder<br>• URL of each funder website<br>• Did the sponsors or funders play any role in the study design, data collection and analysis, decision to publish, or preparation of the manuscript?<br><br>Did you receive funding for this work?	
**Competing Interests**<br><br>Use the instructions below to enter a competing interest statement for this submission. On behalf of all authors, disclose any competing interests that could be perceived to bias this work—acknowledging all financial support and any other relevant financial or non-financial competing interests.<br><br>This statement is required for submission and **will appear in the published article** if the submission is accepted. Please make sure it is accurate and that any funding sources listed in your Funding Information later in the submission form are also declared in your Financial Disclosure statement.<br><br>View published research articles from *PLOS ONE* for specific examples.	The authors declare that there is no conflict of interest.

**NO authors have competing interests**<br><br>Enter: *The authors have declared that no competing interests exist.*<br><br>**Authors with competing interests**<br><br>Enter competing interest details beginning with this statement:<br><br>*I have read the journal's policy and the authors of this manuscript have the following competing interests: [insert competing interests here]*<br><br>* typeset	
**Ethics Statement**<br><br>Enter an ethics statement for this submission. This statement is required if the study involved:<br><br>• Human participants<br>• Human specimens or tissue<br>• Vertebrate animals or cephalopods<br>• Vertebrate embryos or tissues<br>• Field research<br><br>Write "N/A" if the submission does not require an ethics statement.<br><br>General guidance is provided below. Consult the submission guidelines for detailed instructions. **Make sure that all information entered here is included in the Methods section of the manuscript.**	N/A

Powered by Editorial Manager® and ProduXion Manager® from Aries Systems Corporation

**Format for specific study types**<br><br>**Human Subject Research (involving human participants and/or tissue)**<br>• Give the name of the institutional review board or ethics committee that approved the study<br>• Include the approval number and/or a statement indicating approval of this research<br>• Indicate the form of consent obtained (written/oral) or the reason that consent was not obtained (e.g. the data were analyzed anonymously)<br><br>**Animal Research (involving vertebrate animals, embryos or tissues)**<br>• Provide the name of the Institutional Animal Care and Use Committee (IACUC) or other relevant ethics board that reviewed the study protocol, and indicate whether they approved this research or granted a formal waiver of ethical approval<br>• Include an approval number if one was obtained<br>• If the study involved *non-human primates*, add *additional details* about animal welfare and steps taken to ameliorate suffering<br>• If anesthesia, euthanasia, or any kind of animal sacrifice is part of the study, include briefly which substances and/or methods were applied<br><br>**Field Research**<br><br>Include the following details if this study involves the collection of plant, animal, or other materials from a natural setting:<br>• Field permit number<br>• Name of the institution or relevant body that granted permission	
**Data Availability**<br><br>Authors are required to make all data underlying the findings described fully available, without restriction, and from the time of publication. PLOS allows rare exceptions to address legal and ethical concerns. See the PLOS Data Policy and FAQ for detailed information.	Yes - all data are fully available without restriction

A Data Availability Statement describing where the data can be found is required at submission. Your answers to this question constitute the Data Availability Statement and **will be published in the article**, if accepted.<br><br>**Important:** Stating 'data available on request from the author' is not sufficient. If your data are only available upon request, select 'No' for the first question and explain your exceptional situation in the text box.<br><br>Do the authors confirm that all data underlying the findings described in their manuscript are fully available without restriction?	
**Describe where the data may be found in full sentences. If you are copying our sample text, replace any instances of XXX with the appropriate details.**<br><br>• If the data are **held or will be held in a public repository**, include URLs, accession numbers or DOIs. If this information will only be available after acceptance, indicate this by ticking the box below. For example: *All XXX files are available from the XXX database (accession number(s) XXX, XXX.*).<br>• If the data are all contained **within the manuscript and/or Supporting Information files**, enter the following: *All relevant data are within the manuscript and its Supporting Information files.*<br>• If neither of these applies but you are able to provide **details of access elsewhere**, with or without limitations, please do so. For example:<br><br>*Data cannot be shared publicly because of [XXX]. Data are available from the XXX Institutional Data Access / Ethics Committee (contact via XXX) for researchers who meet the criteria for access to confidential data.*<br><br>*The data underlying the results presented in the study are available from (include the name of the third party*	All relevant data are within the manuscript and its Supporting Information files.

and contact information or URL).<br>• This text is appropriate if the data are owned by a third party and authors do not have permission to share the data.<br><br>* typeset	
Additional data availability information:	

Powered by Editorial Manager® and ProduXion Manager® from Aries Systems Corporation

Manuscript Click here to access/download;Manuscript;Manuscript.doc

Effects of core stability exercises on balance ability of children and adolescents with intellectual disabilities: A systematic review and meta-analysis

Junjie Zhou[1], Yecheng Zhong[1], Wenhong Xu[1,*]

[1] College of Physical Education and Health Sciences, Zhejiang Normal University, Jinhua, Zhejiang Province, China

* Corresponding author
E-mail: xuwenhong@zjnu.edu.cn (WX)

// Abstract

Background

In recent years, core stability exercises have been of interest to researchers in improving the balance functions of children and adolescents with disabilities. Studies show that children and adolescents with intellectual disabilities (ID) have more adverse balance and less muscle strength than typically developing peers.

Objective

This systematic review and meta-analysis aim to summarise and quantify the effectiveness of core stability exercises on improving the balance of this target population.

Methods

English-language articles were searched up to June 2023 from 6 electronic databases. Two independent authors assessed the risk of bias using the Cochrane Collaboration's risk of bias tool. Meta-analysis was performed using the RevMan 5.4.

Results

A total of six studies were included, four of which contributed information to the meta-analysis. Our narrative synthesis revealed that core stability exercises improve the dynamic balance of children and adolescents with ID. The pooled analysis shows that core stability exercises were effective for static (Hedges' g: 1.32; 95%CI = -0.41 to 3.06; *p* = .05) and static-dynamic (Hedges' g: 1.35;

95%CI = -0.02 to 2.73; p = .13) balance in children and adolescents with ID, but the effect was insignificant compared with their peers in the control group.

Conclusions

Core stability exercises are effective in improving dynamic balance in children and adolescents with ID. However, no significant effects were found on the improvement in static and static-dynamic balance in this population. More randomized controlled trials are needed to verify the findings of the current study.

Introduction

Balance is a prerequisite to learning complex motor skills during childhood [1], and the foundation of a successful performance of daily and sport-related activities from children to adulthood [2]. Balance is the complex ability to maintain, achieve, or restore the state of equilibrium of the body while an individual stands still, prepares to move, in movement, or prepares to stop moving [3,4]. This capability can be examined in static (the body remains motionless), dynamic (the body reacts to perturbations or is in movement) conditions, or both conditions [5]. Poor balance is a predictor of falling or a failure of results within an activity [6], and always associated with low levels of self-esteem and a sedentary lifestyle, which may lead to obesity, cardiovascular diseases, and high risk of death from all causes [7,8]. Considering that balance performance gradually matures from the age of seven years and plateaus in early adolescence [9,10], attention should be given to the development characteristics and intervention efforts on the balance ability of children and adolescents.

Intellectual disability (ID) is a condition characterized by significant limitations in both intellectual function and adaptive behavior originating before the age of 22 [11]. With an incidence of roughly 2% of the general population [12], ID ranks in the top ten causes of disease burden globally [13]. ID cases are often distinguished as genetic and non-genetic, with Down syndrome (DS), which is caused by the trisomy of *Homo sapiens* chromosome 21, as the most common genomic ID disorder [14]. Children and adolescents with ID usually perform poorer than their typically developing peers in fundamental movement skills [15,16], especially in balance

performance [17]. Indeed, balance deficits among children and adolescents with ID increase the risks of instability, falls, and fall-related injuries, ranging from slight contusion to fractures or even death [5,18].

Studies have shown that individuals balance performance is associated with their core stability [19,20], which is defined as “the ability to achieve and sustain control of the trunk region at rest and during precise movement” [21]. The balance state requires the coordinated work of the core muscles, where the center of gravity of the body’s anatomical position is located [22]. Whether as global (e.g., oblique abdominals and spinalis) or local stabilizers (e.g., multifidus, transverse abdominis, and the pelvic floor) [19], these core stability muscles have a significant effect on a person’s posture by stabilizing the pelvis and the spine and controlling postural fluctuations [23,24]. In fact, research evidence indicates that decreased core stability may predispose individuals to injury resulting from the overcompensation of extremities [25,26], mainly because the core area is central to nearly all kinetic chains of daily activities [20]. Consequently, core stability interventions that aim at improving an individual’s balance ability have aroused the interest of researchers in this area [27,28]. A systematic review and meta-analysis recently reported that core training improves the dynamic balance of athletes and the non-training population, especially among young people [29]. For individuals with disabilities, several studies have provided similar findings [30,31]. The systematic review conducted by Salar et al. [30] revealed that core stability intervention has a positive impact on falling risk among people with visual impairment (VI), regardless of their age, gender, or VI severity. Moreover,

positive intervention effects on balance performance were also observed in children and adolescents with hearing impairment and cerebral palsy [27,28,31]. In recent years, some studies [32,33] have been conducted to understand the effects of core stability exercises on balance ability for children and adolescents with ID. Nevertheless, to date, no systematic review and meta-analysis has yet synthesized and quantified the intervention effects on balance improvement among this population.

To provide new insights for balance improvement in children and adolescents with ID, this systematic review and meta-analysis aim to summarize and quantify the effectiveness of core stability exercises on improving balance in this target population. The findings of our review and meta-analysis can help health professionals, PE teachers, and pediatricians promote balance improvement for this study group. Moreover, research gaps are identified, and guidance is provided for future research in this area.

Methods

Study protocol and registration

This review was registered with the International Prospective Register of Systematic Reviews (PROSPERO; Registration no. CRD42023439091; available from https://www.crd.york.ac.uk/PROSPERO/) and conducted and reported in accordance with the Preferred Reporting Items for Systematic Reviews and Meta-Analysis (PRISMA) statement [34].

Information sources and search strategy

A systematic search of the following electronic databases was performed in June 2023: PubMed, the Web of Science Core Collection, Medline, Embase, Scoups, and the Cochrane Library. The search strategy was agreed upon by JJZ, YCZ and WHX and involved four groups of search terms: (1) intellectual disability, (2) children and adolescents, (3) core stability exercises, and (4) balance. More details about the search strategy are available in S1 Table.

After identifying studies through database search, duplicate records were removed through EndNote X9. Two independent reviewers (JJZ and YCZ) screened the titles and abstracts of all the records retrieved, and full-text articles were obtained for potentially relevant records. Agreement between reviewers was required for the inclusion of full-text articles. Discrepancies were resolved by discussions with a third reviewer (WHX). In addition to database search, the snowballing technique was used to identify potential studies by scanning the reference lists of the included articles.

Inclusion criteria

To be included in the current review, studies must meet the following criteria based on the Population Intervention Comparison Outcome Study design method [35]: (1) population: studies targeting children and adolescents with ID (the sample had a mean age below 18 years); (2) intervention: core stability exercises should be used in the intervention periods; (3) comparison: randomized controlled trials (RCTs) with one or more control groups; (4) outcome: intervention effects on participants' balance ability measured by a standardized tool should be reported in detail; (5) study design: experimental or intervention design was adopted. Additionally, studies must be published in English-language, refereed journal with full-text availability, and only contain original research (i.e., reviews were not considered).

Data extraction

The first author completed the data extraction. Then, the second author checked the data for accuracy. Discrepancies were resolved through a consensus discussion. The descriptive study characteristics (e.g., the first author, the publication year, the geographic location, the recruitment setting, and the sample size), interventional details (i.e., research designs, intervention content, total duration, frequency, and volume), outcome measurements, and results were extracted.

Risk of bias assessment

The Cochrane Collaboration's risk of bias tool was used to evaluate the included RCTs [36]. This tool is valid and reliable and consists of seven domains (i.e., random sequence generation, allocation concealment, blinding of participants and personnel, blinding of outcomes assessment, incomplete outcome data, selective reporting, and

other potential sources of bias) [36]. The appraisal of these domains in each RCT was assessed as having either a high, low, or nuclear risk of bias. The first and second authors independently assessed the risk of bias in the included studies. If consensus could not be reached, agreement was obtained through a discussion with the third author.

Statistical analysis and synthesis

The studies in this review included the effects of core stability exercises on different balance abilities (i.e., static balance, dynamic balance, and static-dynamic balance). If at least two identical outcome variables (e.g., standing time) of the same task (e.g., one leg stance) were available, meta-analysis was performed using the Review Manager software (Version 5.4.1; The Nordic Cochrane Centre, The Cochrane Collaboration, Copenhagen, Denmark). We used the standardized mean difference with a 95% confidence interval (CI) to analyze the effect size. If statistical heterogeneity was found across studies ($I^2 \geq 50\%$, $p < 0.10$), we applied the random-effects model. Otherwise, the fixed-effects model was applied. Hedges' g method was used to reflect the magnitude of the intervention effect [37,38]. According to Cohen et al. [39] a small effect is between ≥ 0.2 and < 0.5, a medium effect was between ≥ 0.5 and < 0.8, and a large effect is ≥ 0.8. Notably, when a study contained two or more control groups, each result was separately analyzed. For those data that could not be synthesized using meta-analyses, we adopted a narrative description of the results across studies.

Results

Search results

A total of 184 studies were identified in the systematic literature search (Web of Science Core Collection, *n* = 44; Scopus, *n* = 61; Medline, *n* = 1; PubMed, *n* = 17; Cochrane Library, *n* = 49; Embase, *n* = 12). These studies were exported to EndNote X9, and 59 duplicates were eliminated. The remaining 125 articles were then screened based on their title and abstract, resulting in the exclusion of 115 studies. In total, 10 studies were reviewed in full text, and six of which were excluded according to the eligibility criteria. Two additional studies were identified by checking the reference lists of the included studies. Ultimately, six primary studies were included. The PRISMA flowchart shows the selection procedure (Fig 1).

Fig 1. The PRISMA flow diagram of the search and screening process.

Risk of bias in included studies

Fig 2 shows the evaluation of the risk of bias in six RCTs. Three studies [40–42] described how random sequences are generated (e.g., computer randomization and the lottery method), and the remaining studies [32,33,43] mentioned “random” but did not explain the specific method they adopted. One study [41] presented a low risk of bias because it reported randomization masking, while five studies [32,33,40,42,43] presented unclear or high risk of bias because they recorded inadequate information about the methods used for allocation concealment. The subjects and personnel were not blinded due to the particularity of the intervention approaches, which made the included trials result in high risk in the performance bias. One study [42] stated

blinding of outcome assessors and was rated as low risk, while the remaining studies [32,33,40,42,43] had an unclear risk of bias. All studies presented a low risk of attrition bias because they reported all data from the study sample. Five studies [32,33,40,42,43] presented a low risk of selective reporting because they included all the measured outcomes, and in one study [41] the presence of selective reporting was unclear.

Fig 2. Risk of bias graph and summary.

Study characteristics

As illustrated in Table 1, three studies were conducted in Iran, followed by Egypt and Pakistan. A total of 173 participants were recorded, with chronological ages varying on average from 7 years to 11 years. The samples were essentially recruited in outpatient clinics or rehabilitation centers (*n* = 4, 66.7%). Three studies [32,33,40] reported the participants' gender and most of them were males. In four of the six studies [40–43], children with DS were recruited. In addition, only three studies [32,33,40] reported ID severity (ranged from mild to moderate).

Table 1 Characteristics of the population in the included studies (*n* = 6).

Study	Country	Recruitment Setting	ID level	Intervention Group			Control Group		
				Sample Size (N)	% of Girls	Mean Age (years)	Sample Size (N)	% of Girls	Mean Age (years)
Ahmadi et al. 2012	Iran	NM	Mild	17	0	11.23±1.95	14	0	11.07±3.02
Alsakhawi & Elshafey, 2019*	Egypt	Outpatient clinic	NM	15	NM	NM	C1: 15<br>C2: 15	C1: NM<br>C2: NM	NM
Aly & Abonour, 2016*	Egypt	Outpatient clinic	Mild-Mod	15	26.7	8.11±1.26	15	33.3	8.34±1.07
Ghaeeni et al. 2015*	Iran	Rehabilitation center	NM	8	NM	9.62±1.68	8	NM	9.87±1.64
Rahmat & Hasan, 2013	Iran	NM	Mild	17	0	11.23±1.95	14	0	11.07±3.02
Zulfiqar et al. 2022*	Pakistan	Rehabilitation center	NM	10	NM	7.30±2.49	10	NM	8.70±3.23

NM, not mentioned; Mod, moderate; C, control group; C1, control group 1; C2, control group 2; *, Participants with Down syndrome.

As illustrated in Table 2, all the studies adopted the RCT design. Core stability exercises based on the Jeffrey protocol were the most used in the six articles included. Except for three studies [32,33,42] that did not report the duration of each exercise, the length of the core stability exercises ranged from 30 min to 60 min per session, for three to four sessions per week and six to eight weeks in total. Five studies [32,33,40–42] presented detailed control conditions and one did not [43]. Finally, none of the studies included a follow-up period.

Table 2 Characteristics of intervention programs, control conditions, and balance measures in the included studies (*n* = 6).

Study	Study design	Intervention				Control condition	Balance measures		
		Content	Total duration (weeks)	Frequency (times/week)	Volume		Types	Measure	Parameters
Ahmadi et al., 2012*	RCT	Abdominal crunch, back extension, hip raise and Russian twist on a stability ball; supine opposite arm-leg raises	6	4	Progressive increase in exercise	Training routines	Dynamic balance	Y TEST	Anterior, Posterolateral and Posteromedial excursion
Alsakhawi & Elshafey, 2019	RCT	30mins traditional exercise program + 30mins core stability exercises based on Jeffrey protocol	8	3	Progressive increase in exercise	C1: 60mins traditional exercise program; C2: 5mins active stretching exercises+30mins traditional exercise program+20mins treadmill exercise program + 5mins cooldown	Static-dynamic balance; Dynamic balance	BBT; BBS	Total Test score; Overall Stability Index
Aly & Abonour, 2016*	RCT	45-60mins core stability exercises based on Jeffrey protocol	8	3	Progressive increase in exercise	Conventional physical therapy program	Dynamic balance	BBS	Overall, anterior-posterior and medial-lateral stability index scores
Ghaeeni et al., 2015	RCT	45-60mins core stability exercises based on Jeffrey protocol	8	3	Progressive increase in exercise	NM	Static balance	MST	Standing time(s)
Rahmat & Hasan, 2013	RCT	Abdominal crunch, back extension, hip	6	4	Progressive increase in	Training routines	Static balance	OLS	Standing time(s)

		raise and Russian twist on a stability ball; supine opposite arm-leg raises			exercise				
Zulfiqar et al., 2022	RCT	Abdominal bracing with or without heel slide in lying position, or leg lifts with same position, same abdominal position with bridging, or standing with abdominal bracing	6	3	NM	Trunk balance exercises	Static-dynamic balance	PBS	Total Test score

NM, not mentioned; RCT, randomized controlled trial; C, control group; C1, control group 1; C2, control group 2; Y TEST, Y Balance Test Protocol; OLS, one leg stance; MST, modified stork test; BBS, Biodex Balance system; BBT, Berg Balance test (scale); PBS, Paediatric berg scale; *, Articles not included in meta-analysis.

Two [33,43] studies assessed static balance through the Modified Stork Test (MST) and the one leg stance test (OLS). Three studies [32,40,41] assessed dynamic balance through the Y Balance Test Protocol (Y TEST) or the Biodex Balance System (BBS). Two studies [41,42] assessed static-dynamic balance through the Berg Balance Test (BBT) or the Paediatric Berg Scale (PBS). In these studies, the measures of balance included the standing time (in seconds) (n = 2), the stability index scores (overall, anterior-posterior, and mediolateral) (n = 2), the limits of stability (anterior, posteromedial, and posterolateral) (n = 1), and the balance scale's score (n = 2).

Effects of Core stability exercises on balance ability

According to different outcome indicators, the included studies were divided into three groups for analysis (i.e., the static, dynamic, and static-dynamic balance groups). The overall results from the studies are presented in Table 3. Compared with the control groups, the studies show that the core stability exercise groups have significantly higher posttest values of dynamic, static, and static-dynamic balance. Within the exercise group, these studies reported a significant improvement (from pretest to posttest) in three groups of balance. In addition, one study found the exercise group had a significantly higher pretest-posttest change values of dynamic balance than the control group.

Table 3 Results reported for outcomes of included studies (*n* = 6).

Study	IG (Pretest vs Posttest)	CG (Pretest vs Posttest)	IG vs CG (Posttest)	IG vs CG (Pretest-Posttest Change)
Ahmadi et al., 2012	Improvement (only for posterolateral and posteromedial excursion)	NS	IG > CG (only for posterolateral and posteromedial excursion)	NM
Alsakhawi & Elshafey, 2019	Improvement (all)	Improvement (all)	IG > CG1(all); NS (IG vs CG2)	NM
Aly & Abonour, 2016	Improvement (all)	Improvement (all)	IG > CG (all)	IG > CG (all)
Ghaeeni et al., 2015	Improvement	NS	IG > CG	NM
Rahmat & Hasan, 2013	Improvement	NM	IG > CG	NM
Zulfiqar et al., 2022	Improvement	NS	IG > CG	NM

CG, control group; CG1, control group 1; CG2, control group 2; IG, intervention group; NM, not mentioned; NS, not significant.

Dynamic balance

As different balance parameters were used, we opted to narratively describe the results of the dynamic balance [32,40,41]. Two studies [40,41] that used BBS concluded that core stability exercises effectively improve the overall stability index scores in children and adolescents with DS. Additionally, one study [32] used the Y TEST as measurement tool and found that participants in the intervention group had a significant improvement in mean posterolateral and posteromedial excursion in the post-test ($p \leq .05$), but that in the anterior direction is insignificant ($p \leq .27$).

Static balance

Static balance was assessed in two RCTs [33,43] and the data were pooled (Fig 3). The OLS and the MST were used to measure static balance in a total of 47 ID participants. The overall pooled effect size of the static balance function in the experimental group did not exhibit significant enhancement compared with the control [Hedges' g: 1.32 (95%CI: -0.41, 3.06) at $p = .13$], with a highly significant difference in heterogeneity (I^2= 81% at $p = .02$).

Fig 3. Forest plot for the effect of core stability exercise on static balance.

Static-dynamic balance

Two studies used the BBT and PBS to investigate participants' static-dynamic balance ability [41,42]. We analyzed 3 effect sizes for one study had two control groups (Fig 4). The overall pooled effect size of the static-dynamic balance function in the experimental group did not exhibit significant enhancement compared with the control [Hedges' g: 1.35 (95%CI: -0.02, 2.73) at $p = .05$]. I^2 indicated a high level of heterogeneity (I^2 = 86% at $p < .001$).

Fig 4. Forest plot for the effect of core stability exercise on static-dynamic balance.

Discussion

This systematic review and meta-analysis aim to assess the effects of core stability exercises on the balance ability of children and adolescents with ID. The results of our review show that core stability exercises significantly improved the dynamic balance in this target population (Table 3). For static and static-dynamic balance, core stability exercises tend to be effective in individuals with ID. However, the effects are insignificant compared with the effect on their peers in the control groups (Figs 3 and 4).

Dynamic balance refers to the subject's ability to react efficiently to the base of support displacements [44]. The present review suggests that core stability exercises are beneficial for dynamic balance ability in children and adolescents with ID, which is consistent with the athletes and non-trained populations of core stability exercises intervention review studies [29]. Given the limits of intellectual function, children and adolescents with ID usually experience delays in area of cognitive development and motor function [45]. Many studies have found that sedentary lifestyles and obesity are highly prevalent in children and youth with ID compared with typically developing peers [46,47], which in turn further hinders physical activity participation and balance development. According to Anderson et al. [22] balance is achieved through an interaction between central anticipatory and reflexive actions, and these actions are assisted by the active and passive restraints caused by the muscular system. Factors that influence balance include the sensory information obtained from the

somatosensory, visual, and vestibular system and motor responses that affect coordination, the joint range of motion, and strength [48]. Repetitive training experiences that influence motor responses and the earlier activation of core muscles by the central nervous system in anticipation of movements may be the underlying mechanism of balance improvement [29]. Meanwhile, complex environmental changes (e.g., visual or motor) during training may beneficial for sensori-motor systems in children and adolescents in ID. Additionally, core stability exercises can improve the hip and trunk muscle strength, which help build a solid, stable base and is important to increase dynamic balance, especially in children and youth with ID with relatively weak muscle strength [49]. The maintenance and recovery of balance is possible due to the coordinated work of core muscles where the center of gravity of the body’s anatomical position is located [20,22]. The most used exercise in the articles included is abdominal crunch, which recruits deep muscles of the spine, such as the lumbar multifidus, and abdominal muscles, such as the transversus abdominis [50]. These deep muscles of the trunk support the spine and maintain the dynamic balance of the lower extremity [20,40].

The results of this review show that core stability exercises had a positive effect on the dynamic balance in participants with a mean age of 7–11 years. The majority of the samples were children, which may be because researchers focused more on the most critical period of motor development, that is, the first decade of life [51,52]. However, plateaus of balance are usually in early adolescence (i.e., 12–14 years) at which it is largely equivalent to average adult performance [10,53]. That is to say,

balance still needs attention at the teenage years [54]. During the adolescent growth spurt, a differentiation exists between fast bone growth and low growth of musculature, coupled with a decrease in tendons flexibility [55]. For adolescents with ID, increasing body dimensions and changing proportions can be a challenge when they are shaping skills to maintain body balance [56]. Therefore, future studies should pay more attention to adolescents with ID to observe the results of the interventions aiming to improve the balance ability.

The present review also show that a common feature of the interventions was the progressive increase of the volume in core stability exercises. The progression of the exercise training load is one of the main training principles [57]. A previous study conducted by Vera-Garcia et al. [58] revealed that the progression of exercises based on individual’s development and the movement pattern learned seem to be more decisive for dynamic balance performance. In the current review, three studies [32,40,41] on improved dynamic balance all used increased the volume progressively during the core stability exercises. Two of these [40,41] used the Jeffery protocol [59], which consists of exercises of progressively increasing difficulty that focus on strengthening the abdominal, lower back, and pelvic muscles. This result is consistent with previous findings in different populations that dynamic balance increased after progressive exercises [29,60,61].

In terms of the instruments for the evaluation of the outcomes, two studies [40,41] that used BBS concluded that core stability exercises effectively improve the overall stability index score in children and adolescents with DS. The overall stability index

score is believed to be the best indicator of the overall ability to balance the platform [62]. As the tool for measuring dynamic balance, Ahmadi et al. [32] using the Y TEST assessed three types of movements, namely, anterior, posteromedial, and posterolateral. The lower quarter Y TEST (using only 3 axes of the star excursion balance test: anterior, posteromedial, and posterolateral) is a common method of assessing dynamic balance [29]. The interesting finding is that the core stability exercise improved the posteromedial and posterolateral excursion, but was insignificant in the anterior direction. Electromyographic activity in the lower limbs has been reported to be direction-dependent in the star offset balance test, with higher activation in certain directions than in others [63], indicating that core stability exercises have few effects in the anterior direction for neuromuscular system control and muscle strengthening [32].

The quantitative results show that core stability exercises have no significant effect on static (p = .13) and static-dynamic (p = .05) balance in participants with ID than those in the control groups. Meanwhile, significant heterogeneity exists in the results of both our meta-analyses. This outcome could be caused by three factors. First, the numbers of studies and participants included in the analysis are relatively small. Second, the characteristics of the participants may vary. Lipowicz et al. [56] pointed out that moderate disability is a factor that significantly reduces the ability to maintain the balance of children and youth with ID. However, among the included studies, only one study [33] reported the ID severity of the participants. Third, although most of the core stability exercises have a unified training content, the

included research may cover inconsistent training approaches to conduct intervention, such as the instruction technique or supervision process. Given the limited number of studies and their heterogeneity, more research is needed in the future to precisely estimate the effectiveness of core stability exercises on static and static-dynamic balance.

Limitations

Among the six selected studies, five [32,33,40,41,43] were conducted in the Eastern Mediterranean region. Thus, the findings may not be generalizable to other nations and ethnicities. Another limitation is the probability of publication bias, which we attempted to decrease via a substantial database search. Due to the language barriers and resource limitations, however, only studies published in English were included. Studies published in national languages in Asia and other countries were not included in this review. Finally, a limited number of studies were included in this review and even though their similar characteristics, they remain heterogeneous in their interventions, control conditions, and the characteristics of the participant.

Implications

Despite the limitations, implications for future research can be derived from the present review. First, although the review studies were RCTs, most of the research failed to demonstrate concealed allocation, the blinding of participants and assessors, which are all required to substantiate evidence of the strong efficacy of an intervention [64]. Meanwhile, the included studies lack strict study designs and adequate sample size to optimize the power of the statistical analysis, which could

affect the maturity of the measured outcomes. Second, core stability exercises were only conducted for a short period (6–8 weeks), which is insufficient to support the physical and cognitive integration of new skills to achieve the long-term modification of balance ability in children and adolescents with ID [65]. Planning an appropriate duration to promote the full realization of each child’s balance potential may be more helpful. Third, future studies need to provide sufficient details about their exercise interventions, such as their settings, the facilitator (i.e., the person who delivers the intervention), and the adaptation of the intervention during the study.

Conclusions

The present results show that core stability exercises are an efficient means to improving dynamic balance in children and adolescents with ID. However, the currently limited data suggests no significant effect on the improvement of static and static-dynamic balance in children and adolescents with ID. Therefore, further rigorous studies are needed to strengthen the evidence.

References

1. Mickle KJ, Munro BJ, Steele JR. Gender and age affect balance performance in primary school-aged children. Journal of Science and Medicine in Sport. 2011; 14(3): 243–8.
2. Gebel A, Lesinski M, Behm DG, Granacher U. Effects and Dose–Response Relationship of Balance Training on Balance Performance in Youth: A Systematic Review and Meta-Analysis. Sports Med. 2018; 48(9): 2067–89.
3. Melo RS, Tavares-Netto AR, Delgado A, Wiesiolek CC, Ferraz KM, Belian RB. Does the practice of sports or recreational activities improve the balance and gait of children and adolescents with sensorineural hearing loss? A systematic review. Gait & Posture. 2020; 77: 144–55.
4. Pollock AS, Durward BR, Rowe PJ, Paul JP. What is balance? Clin Rehabil. 2000; 14(4): 402–6.
5. Paillard T. Plasticity of the postural function to sport and/or motor experience. Neuroscience & Biobehavioral Reviews. 2017; 72: 129–52.
6. Almuhtaseb S, Oppewal A, Hilgenkamp TIM. Gait characteristics in individuals with intellectual disabilities: A literature review. Research in Developmental Disabilities. 2014; 35(11): 2858–83.
7. Cao C, Cade WT, Li S, McMillan J, Friedenreich C, Yang L. Association of Balance Function With All-Cause and Cause-Specific Mortality Among US Adults. JAMA Otolaryngology–Head & Neck Surgery. 2021; 147(5): 460–8.

8. Nofuji Y, Shinkai S, Taniguchi Y, Amano H, Nishi M, Murayama H, et al. Associations of Walking Speed, Grip Strength, and Standing Balance With Total and Cause-Specific Mortality in a General Population of Japanese Elders. Journal of the American Medical Directors Association. 2016; 17(2): 184.e1-184.e7.

9. Blodgett JM, Cooper R, Pinto Pereira SM, Hamer M. Stability of Balance Performance From Childhood to Midlife. Pediatrics. 2022; 150(1): e2021055861.

10. Schedler S, Kiss R, Muehlbauer T. Age and sex differences in human balance performance from 6-18 years of age: A systematic review and meta-analysis. Neto MG, editor. PLoS ONE. 2019; 14(4): e0214434.

11. Schalock R, Luckasson R, Tassé M. An Overview of Intellectual Disability: Definition, Diagnosis, Classification, and Systems of Supports (12th ed.). American Journal on Intellectual and Developmental Disabilities. 2021; 126: 439–42.

12. McBride O, Heslop P, Glover G, Taggart T, Hanna-Trainor L, Shevlin M, et al. Prevalence estimation of intellectual disability using national administrative and household survey data: The importance of survey question specificity. Int J Popul Data Sci. 2021; 6(1): 1342.

13. Totsika V, Liew A, Absoud M, Adnams C, Emerson E. Mental health problems in children with intellectual disability. The Lancet Child & Adolescent Health. 2022; 6(6): 432–44.

14. Antonarakis SE, Skotko BG, Rafii MS, Strydom A, Pape SE, Bianchi DW, et al. Down syndrome. Nat Rev Dis Primers. 2020; 6(1): 9.

15. Kavanagh H, Manninen M, Issartel J. Comparing the fundamental movement skill proficiency of children with intellectual disabilities and typically developing children: a systematic review and meta-analysis. J intellect Disabil Res. 2023; 67(12): 1336-1353.

16. Li X, Huang J, Kong Z, Sun F, Sit CHP, Li C. Effects of Virtual Reality-Based Exercise on Physical Fitness in People with Intellectual Disability: A Systematic Review of Randomized Controlled Trials. Games for Health Journal. 2023; 12(2): 89–99.

17. Sretenović I, Nedović G, Đorđević S. Assessment of balance in younger school age children with intellectual disability. FU Phys Ed Sport. 2019; 687-696.

18. Matson JL, Matson ML, editors. Comorbid Conditions in Individuals with Intellectual Disabilities. Cham: Springer International Publishing; 2015.

19. Borghuis J, Hof AL, Lemmink KAPM. The Importance of Sensory-Motor Control in Providing Core Stability. Sports Med. 2008; 38(11): 893–916.

20. Kibler WB, Press J, Sciascia A. The Role of Core Stability in Athletic Function. Sports Med. 2006; 36(3): 189–98.

21. Majewski-Schrage T, Evans TA, Ragan B. Development of a Core-Stability Model: A Delphi Approach. Journal of Sport Rehabilitation. 2014; 23(2): 95–106.

22. Anderson K, Behm DG. The Impact of Instability Resistance Training on

Balance and Stability. Sports Med. 2005; 35(1): 43–53.

23. Arnold C, Lanovaz J, Oates A, Craven B, Butcher S. The effect of adding core stability training to a standard balance exercise program on sit to stand performance in older adults: a pilot study. J Aging Phys Act. 2015; 23(1): 95–102.

24. Sadeghi H, Shojaedin SS, Alijanpour E, Abbasi A. The Effects of Core Stability Exercises on Balance and Walking in Elderly Fallers with Mild Cognitive Impairment: A Randomized Control Trial. Journal of Research in Rehabilitation of Sciences. 2020; 16: 110–7.

25. Oliver G, Adams-Blair H, Dougherty C. Implementation of a Core Stability Program for Elementary School Children. Athletic Training & Sports Health Care. 2010; 2(6): 261-6.

26. Willson JD, Dougherty CP, Ireland ML, Davis IM. Core Stability and Its Relationship to Lower Extremity Function and Injury: Journal of the American Academy of Orthopaedic Surgeons. 2005; 13(5): 316–25.

27. Ali MS, Awad AS, Elassal MI. The effect of two therapeutic interventions on balance in children with spastic cerebral palsy: A comparative study. Journal of Taibah University Medical Sciences. 2019; 14(4): 350–6.

28. Elshafey MA, Abdrabo MS, Elnaggar RK. Effects of a core stability exercise program on balance and coordination in children with cerebellar ataxic cerebral palsy. Journal of Musculoskeletal and Neuronal Interactions. 2022; 22(2): 172.

29. Barrio ED, Ramirez-Campillo R, Garcia de Alcaraz Serrano A, RaquelHernandez-García R. Effects of core training on dynamic balance stability: A systematic review and meta-analysis. Journal of Sports Sciences. 2022; 40(16): 1–9.

30. Salar S, Ardakani M, Lieberman L, Beach P, Perreault M. The effects of balance and core stability training on postural control in people with visual impairment: A systematic review. British Journal of Visual Impairment. 2022;026461962210772.

31. Zarei H, Norasteh AA. Effects of proprioception and core stability training followed by detraining on balance performance in deaf male students: a three-arm randomized controlled trial. Somatosens Mot Res. 2023; 40(2): 47–55.

32. Ahmadi R, Hasan D, Hosin BA. The effect of 6 weeks core stabilization training program on the balance in mentally retarded students. Medicina Sportiva. 2012; 8(4): 2003-2008.

33. Rahmat A, Hasan D. The effect of core stabilization exercises on factors physical fitness to mental retardation. Medicina Sportiva. 2013; 9(1): 2058-2062.

34. Page MJ, McKenzie JE, Bossuyt PM, Boutron I, Hoffmann TC, Mulrow CD, et al. The PRISMA 2020 statement: an updated guideline for reporting systematic reviews. Systematic Reviews. PLoS Med. 2021; 10(1): 89.

35. Amir-Behghadami M, Janati A. Population, Intervention, Comparison, Outcomes and Study (PICOS) design as a framework to formulate eligibility

criteria in systematic reviews. Emerg Med J. 2020; 37(6): 387–387.

36. Higgins JPT, Altman DG, Gotzsche PC, Juni P, Moher D, Oxman AD, et al. The Cochrane Collaboration’s tool for assessing risk of bias in randomised trials. BMJ. 2011; 343.

37. Wasserman S, Hedges LV, Olkin I. Statistical Methods for Meta-Analysis. Journal of Educational Statistics. 1985; 13(1): 75.

38. Hedges LV. Distribution Theory for Glass’s Estimator of Effect size and Related Estimators. Journal of Educational Statistics. 1981; 6(2): 107–28.

39. Cohen J. Statistical power analysis for the behavioral sciences. 2nd ed. Hillsdale, N.J.: Lawrence Erlbaum Associates; 1988.

40. Aly SM, Abonour AA. Effect of core stability exercise on postural stability in children with Down syndrome. International Journal of Medical Research & Health Sciences. 2016; 5: 213-22.

41. Alsakhawi RS, Elshafey MA. Effect of Core Stability Exercises and Treadmill Training on Balance in Children with Down Syndrome: Randomized Controlled Trial. Adv Ther. 2019; 36(9): 2364–73.

42. Zulfiqar H, Rehman HMU, Razzaq A, Nisa ZU, Hina M, Bashir H, et al. Effect Of Core Stability Exercises and Balance Training in Postural Control Among Children with Down Syndrome : Stability Exercises and Balance Training in Postural Control Among Children. Pakistan BioMedical Journal. 2022; 5(7): 18–22.

43. Ghaeeni S, Bahari Z, Khazaei AA. Effect of Core Stability Training on Static

Balance of the Children with Down Syndrome. Physical Treatments-Specific Physical Therapy Journal. 2015; 5(1): 49-53.

44. Paillard T. Relationship Between Sport Expertise and Postural Skills. Front Psychol. 2019; 10: 1428.

45. Hocking J, McNeil J, Campbell J. Physical therapy interventions for gross motor skills in people with an intellectual disability aged 6 years and over: a systematic review. International Journal of Evidence-Based Healthcare. 2016; 14(4): 166–74.

46. Wang J, Gao Y, Kwok HHM, Huang WYJ, Li S, Li L. Children with Intellectual Disability Are Vulnerable to Overweight and Obesity: A Cross-Sectional Study among Chinese Children. Childhood Obesity. 2018; 14(5): 316–26.

47. Yuan Y, Ding J, Wang C, Zhang S, Wang Y, Liu Y, et al. The after-school sedentary behavior status among children and adolescents with intellectual disabilities. Front Psychiatry. 2022; 13: 1049180.

48. Palmieri RM, Ingersoll CD, Cordova ML, Kinzey SJ, Stone MB, Krause BA. The effect of a simulated knee joint effusion on postural control in healthy subjects11No commercial party having a direct financial interest in the results of the research supporting this article has or will confer a benefit upon the author(s) or upon any organization with which the author(s) is/are associated. Archives of Physical Medicine and Rehabilitation. 2003; 84(7): 1076–9.

49. Sadeghi H, Shariat A, Asadmanesh E, Mosavat M. The Effects of Core

Stability Exercise on the Dynamic Balance of Volleyball Players. International Journal of Applied Exercise Physiology. 2013; 2(2): 1-10.

50. Hodges PW. Is there a role for transversus abdominis in lumbo-pelvic stability? Manual Therapy. 1999; 4(2): 74–86.

51. Ferdjallah M, Harris GF, Smith P, Wertsch JJ. Analysis of postural control synergies during quiet standing in healthy children and children with cerebral palsy. Clinical Biomechanics. 2002; 17(3): 203–10.

52. Roncesvalles MNC, Woollacott MH, Jensen JL. Development of Lower Extremity Kinetics for Balance Control in Infants and Young Children. Journal of Motor Behavior. 2001; 33(2): 180–92.

53. Peterson ML, Christou E, Rosengren KS. Children achieve adult-like sensory integration during stance at 12-years-old. Gait & Posture. 2006; 23(4): 455–63.

54. Viel S, Vaugoyeau M, Assaiante C. Adolescence: A Transient Period of Proprioceptive Neglect in Sensory Integration of Postural Control. Motor Control. 2009; 13(1): 25–42.

55. Wild CY, Steele JR, Munro BJ. Musculoskeletal and Estrogen Changes during the Adolescent Growth Spurt in Girls. Medicine & Science in Sports & Exercise. 2013; 45(1): 138–45.

56. Lipowicz A, Bugdol MN, Szurmik T, Bibrowicz K, Kurzeja P, Mitas AW. Body balance analysis of children and youth with intellectual disabilities. Journal of Intellectual Disability Research. 2019; 63(11): 1312–23.

57. Kasper K. Sports Training Principles: Current Sports Medicine Reports. 2019;

18(4): 95–6.

58. Vera-Garcia FJ, Irles-Vidal B, Prat-Luri A, García-Vaquero MP, Barbado D, Juan-Recio C. Progressions of core stabilization exercises based on postural control challenge assessment. Eur J Appl Physiol. 2020; 120(3): 567–77.

59. Jeffreys I. Developing a Progressive Core Stability Program: Strength and Conditioning Journal. 2002; 24(5): 65–6.

60. Bashir SF, Nuhmani S, Dhall R, Muaidi QI. Effect of core training on dynamic balance and agility among Indian junior tennis players. BMR. 2019; 32(2): 245–52.

61. Imai A, Kaneoka K, Okubo Y, Shiraki H. Effects of two types of trunk exercises on balance and athletic performance in youth soccer players. Int J Sports Phys Ther. 2014; 9(1): 47–57.

62. Aydoğ E, Depedibi R, Bal A, Ekşioğlu E, Ünlü E, Çakci A. Dynamic postural balance in ankylosing spondylitis patients. Rheumatology. 2006; 45(4): 445–8.

63. Earl JE, Hertel J. Lower-Extremity Muscle Activation during the Star Excursion Balance Tests. Journal of Sport Rehabilitation. 2001; 10(2): 93–104.

64. Higgins JPT, Green S, Cochrane Collaboration, editors. Cochrane handbook for systematic reviews of interventions. Chichester, England ; Hoboken, NJ: Wiley-Blackwell; 2008. 649 p. (Cochrane book series).

65. Maïano C, Hue O, Morin AJS, Lepage G, Tracey D, Moullec G. Exercise interventions to improve balance for young people with intellectual disabilities: a systematic review and meta-analysis. Dev Med Child Neurol. 2019; 61(4):

406–18.

Supporting information

S1 Table. Details of search query.

S2 Table. PRISMA 2020 Checklist.

Fig 1. The PRISMA flow diagram of the search and screening process.

Click here to access/download;Figure;Fig1.eps

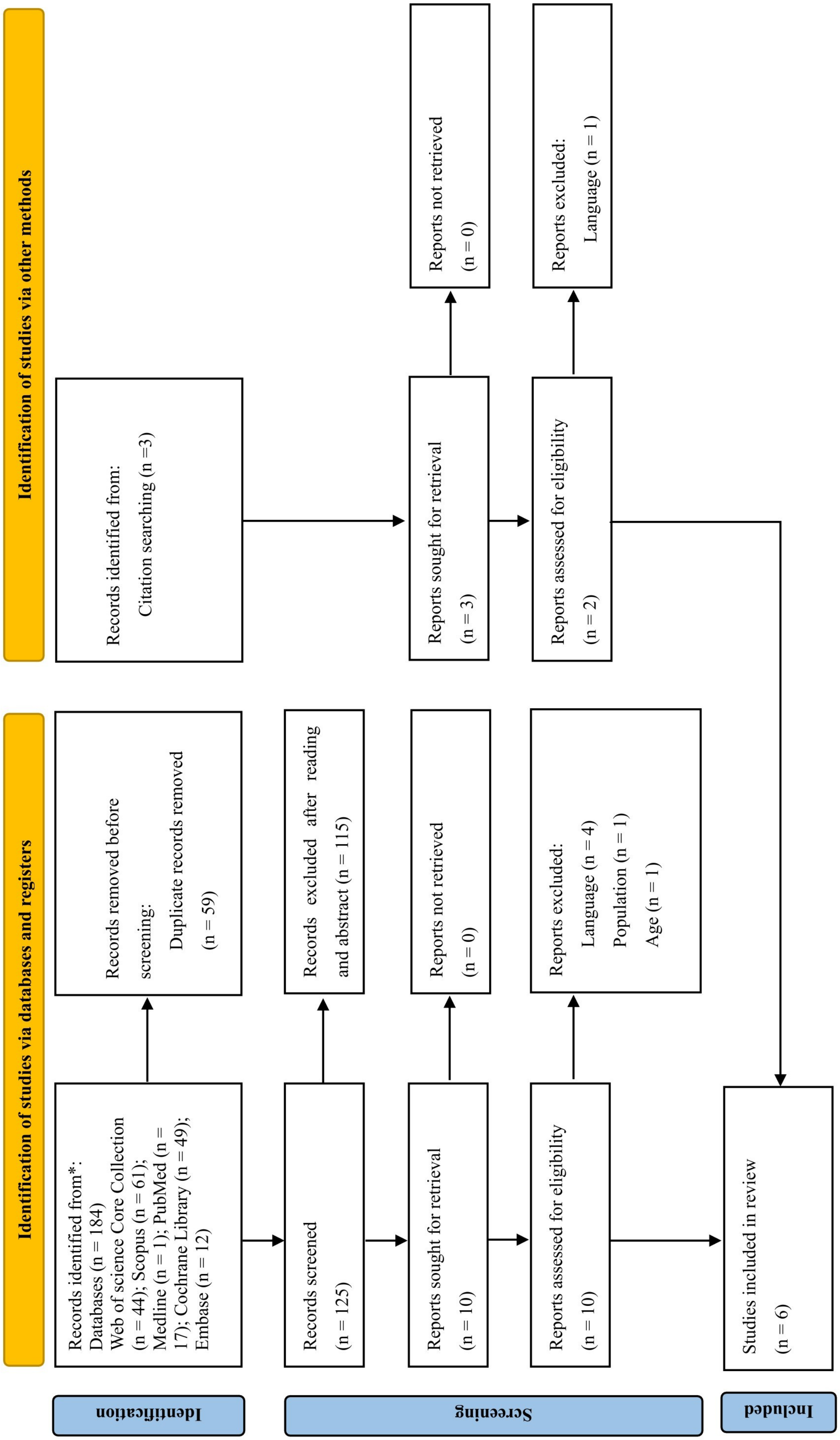

Fig 2. Risk of bias graph and summary.

Click here to access/download;Figure;Fig2.eps

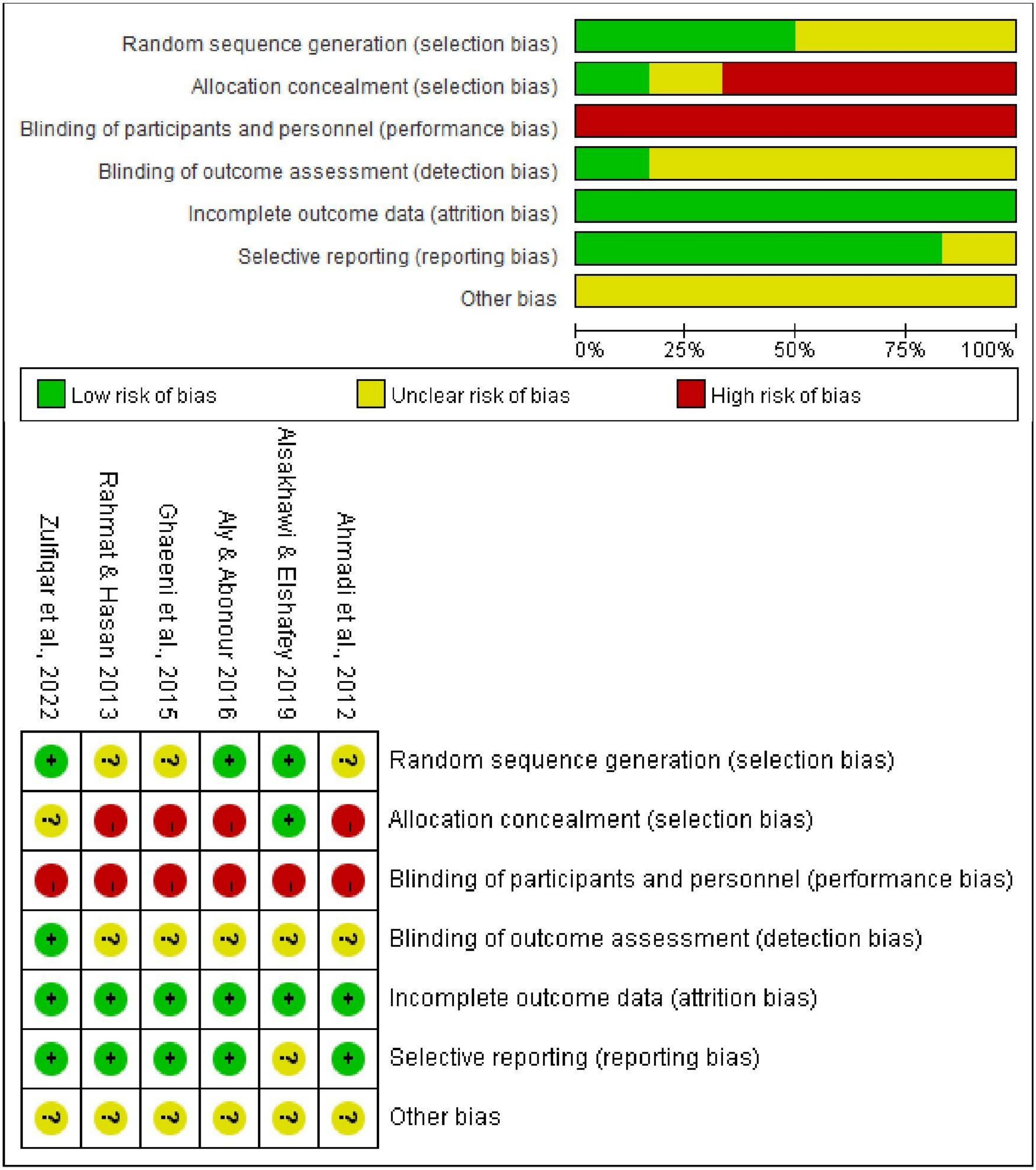

Fig 3. Forest plot for the effect of core stability exercise on static balance.

Click here to access/download;Figure;Fig3.eps

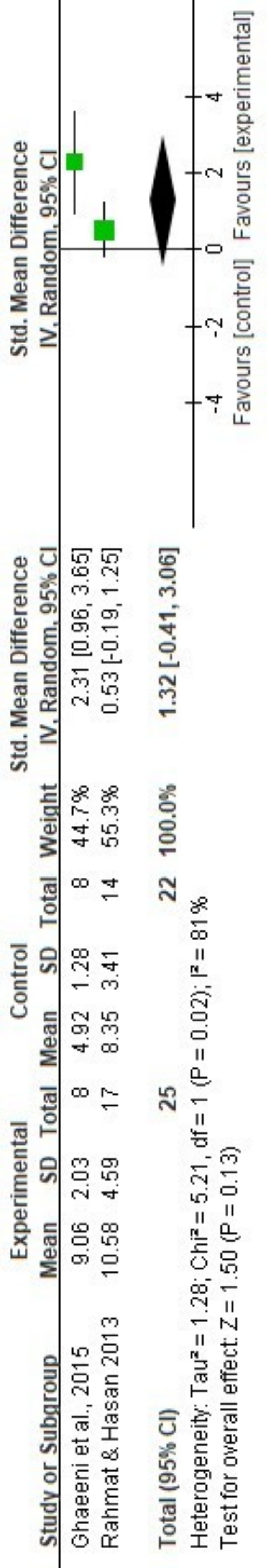

Fig 4. Forest plot for the effect of core stability exercise on static-dynamic balance.

Click here to access/download;Figure;Fig4.eps

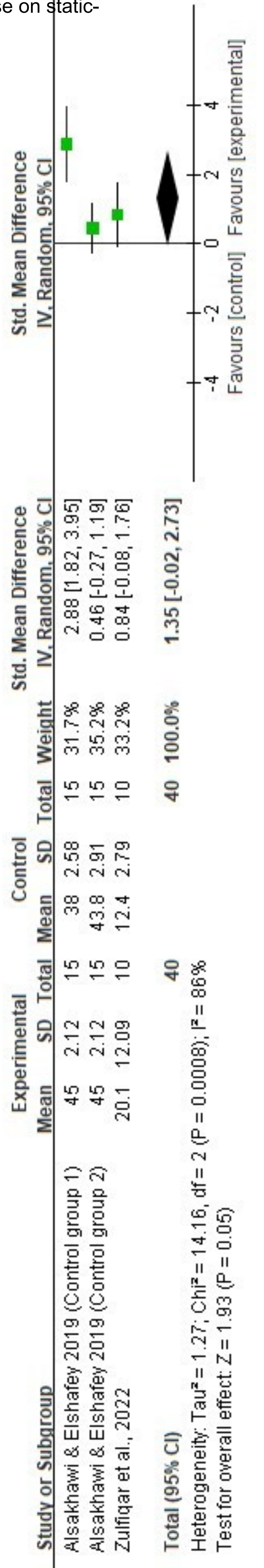

S1 Details of search query

Click here to access/download
Supporting Information
S1 Table. Details of search query..docx

S2 PRISMA 2020 Checklist

Click here to access/download

Supporting Information

S2 Table. PRISMA 2020 Checklist..docx
