# Supplementary material for: Effects of core stability exercises on balance ability of children and adolescents with intellectual disabilities: A systematic review and meta-analysis
Source: PLoS One. 2024 Dec 19;19(12):e0314664. doi: 10.1371/journal.pone.0314664 (PMC11658597; doi:10.1371/journal.pone.0314664)
Supplement: Attachment — Submitted filename: PLOS one.docx [file pone.0314664.s004.docx]

The paper “Effects of core stability exercises on balance ability of children and adolescents with intellectual disabilities: A systematic review and meta-analysis” is a well-written paper but there are some information lacking.

I propose some change and reflections;

General:

Please explain the acronyms before putting them in the text.

Inclusion criteria: please insert more information about etiology of the patient. Neuromuscolar disease have been excluded? Motor disease have been excluded?

The article selected are too inconsistent with each other. Please insert more documentation about ID. ID is name only in general, no specific determination about language, performance of ID are explained.

Please insert information about tools used to performed intelligence quotient.

Specific:

Line 58-59- Intellectual disability (ID) is a condition characterized by significant limitations

59 in both intellectual function and adaptive behavior originating before the age of 22. I would ask to the author to chek if the age is 22 and not 18. In line 127 studies authors said that they targeting children and adolescents mean age below 18 years.

63: the author refers to Down Syndrome ad the most common cause of ID, please specify differences between male and female

67: How can author divided between fall andr critical falls, in other words due to epileptic issues?
