# Supplementary material for: Effects of core stability exercises on balance ability of children and adolescents with intellectual disabilities: A systematic review and meta-analysis
Source: PLoS One. 2024 Dec 19;19(12):e0314664. doi: 10.1371/journal.pone.0314664 (PMC11658597; doi:10.1371/journal.pone.0314664)
Supplement: Attachment — Submitted filename: Response to reviewers.doc [file pone.0314664.s007.doc]

Dear reviewers:

Thank you once again for your constructive comments on our revised manuscript titled “Effects of core stability exercises on balance ability of children and adolescents with intellectual disabilities: A systematic review and meta-analysis” [PONE-D-24-21189R1]. Your insights have been invaluable in refining our work, and we have carefully addressed each of your suggestions in this second round of revisions. The main corrections are in the manuscript and the response to the reviewers’ comments are as follows (the replies are highlighted in bold).

Replies to the reviewers’ comments:

Reviewer #2: I congratulate the authors as the new version of the manuscript presents substantial improvements. All my initial concerns were addressed by the authors in the first review of the manuscript. The conclusion described in the abstract ‘Core stability exercises appear to be a promising and acceptable intervention for the improvement of dynamic balance in children and adolescents with ID’ seems abusive to me, considering the scarcity of studies included, the results of the studies and the certainty of the evidence synthesised. In order to be published, the manuscript must be reviewed idiomatically and scientifically in terms of writing style.

**Response: Thank you for your positive comments.** **We have revised the conclusion section of the abstract.** **The revised text reads: “Core stability exercises may improve dynamic balance in children and adolescents with ID, but given the scarcity of studies included, definitive conclusions cannot yet be drawn.” (Page 3, lines 39-41).** **Our manuscript has been meticulously edited by professional editors from AJE’s editing team to ensure correct English language, grammar, punctuation, spelling, and overall style (you can verify this using the verification code 5833-2A66-21B7-AB08-9768 on the AJE website).**

Reviewer #5:

Well done on putting this manuscript together.

You have addressed all my comments very well. All the best

**Response:** **Thank you very much for your encouragement. We are glad to hear that we have effectively addressed your comments. Your thorough review and constructive feedback have been instrumental in improving our manuscript. We appreciate your time and effort and wish you all the best as well.**

Journal Requirements: Please review your reference list to ensure that it is complete and correct. If you have cited papers that have been retracted, please include the rationale for doing so in the manuscript text, or remove these references and replace them with relevant current references. Any changes to the reference list should be mentioned in the rebuttal letter that accompanies your revised manuscript. If you need to cite a retracted article, indicate the article’s retracted status in the References list and also include a citation and full reference for the retraction notice.

**Response: We have thoroughly reviewed and checked our list of references in accordance with the requirements of your journal, ensuring its completeness and accuracy. As part of this process, we verified and updated, when applicable, unique identifiers such as DOI and PMID to maintain the accuracy and accessibility of each reference. Furthermore, we did not include any references to articles that have been retracted (Page 31-44).**

Kind regards

Sincerely,

Junjiie Zhou (The First Author)

E-mail: [zhoujunjie@zjnu.edu.cn](mailto:zhoujunjie@zjnu.edu.cn)

Wenhong Xu (Corresponding author)

E-mail: [xuwenhong@zjnu.edu.cn](mailto:xuwenhong@zjnu.edu.cn)

04 October, 2024

College of Physical Education and Health Sciences

Zhejiang Normal University

Jinhua, China
